# Genome-Wide Analysis of DNA Methylation and Fine Particulate Matter Air Pollution in Three Study Populations: KORA F3, KORA F4, and the Normative Aging Study

**DOI:** 10.1289/ehp.1509966

**Published:** 2016-01-05

**Authors:** Tommaso Panni, Amar J. Mehta, Joel D. Schwartz, Andrea A. Baccarelli, Allan C. Just, Kathrin Wolf, Simone Wahl, Josef Cyrys, Sonja Kunze, Konstantin Strauch, Melanie Waldenberger, Annette Peters

**Affiliations:** 1 Institute of Epidemiology II, Helmholtz Zentrum München, German Research Center for Environmental Health, Neuherberg, Germany; 2 Department of Environmental Health, and; 3 Laboratory of Environmental Epigenetics, Exposure Epidemiology and Risk Program, Harvard T.H. Chan School of Public Health, Boston, Massachusetts, USA; 4 Research Unit of Molecular Epidemiology, and; 5 Institute of Genetic Epidemiology, Helmholtz Zentrum München, German Research Center for Environmental Health, Neuherberg, Germany; 6 Department of Genetic Epidemiology, Institute of Medical Informatics, Biometry and Epidemiology, Ludwig-Maximilians-Universität, Munich, Germany; 7 German Research Center for Cardiovascular Disease (Deutsches Zentrum Für Herz-Kreislauf-Forschung E.V.), Munich, Germany

## Abstract

**Background:**

Epidemiological studies have reported associations between particulate matter (PM) concentrations and cancer and respiratory and cardiovascular diseases. DNA methylation has been identified as a possible link but so far it has only been analyzed in candidate sites.

**Objectives:**

We studied the association between DNA methylation and short- and mid-term air pollution exposure using genome-wide data and identified potential biological pathways for additional investigation.

**Methods:**

We collected whole blood samples from three independent studies—KORA F3 (2004–2005) and F4 (2006–2008) in Germany, and the Normative Aging Study (1999–2007) in the United States—and measured genome-wide DNA methylation proportions with the Illumina 450k BeadChip. PM concentration was measured daily at fixed monitoring stations and three different trailing averages were considered and regressed against DNA methylation: 2-day, 7-day and 28-day. Meta-analysis was performed to pool the study-specific results.

**Results:**

Random-effect meta-analysis revealed 12 CpG (cytosine-guanine dinucleotide) sites as associated with PM concentration (1 for 2-day average, 1 for 7-day, and 10 for 28-day) at a genome-wide Bonferroni significance level (p ≤ 7.5E-8); 9 out of these 12 sites expressed increased methylation. Through estimation of I2 for homogeneity assessment across the studies, 4 of these sites (annotated in NSMAF, C1orf212, MSGN1, NXN) showed p > 0.05 and I2 < 0.5: the site from the 7-day average results and 3 for the 28-day average. Applying false discovery rate, p-value < 0.05 was observed in 8 and 1,819 additional CpGs at 7- and 28-day average PM2.5 exposure respectively.

**Conclusion:**

The PM-related CpG sites found in our study suggest novel plausible systemic pathways linking ambient PM exposure to adverse health effect through variations in DNA methylation.

**Citation:**

Panni T, Mehta AJ, Schwartz JD, Baccarelli AA, Just AC, Wolf K, Wahl S, Cyrys J, Kunze S, Strauch K, Waldenberger M, Peters A. 2016. A genome-wide analysis of DNA methylation and fine particulate matter air pollution in three study populations: KORA F3, KORA F4, and the Normative Aging Study. Environ Health Perspect 124:983–990; http://dx.doi.org/10.1289/ehp.1509966

## Introduction

Ambient air pollution has been associated with total mortality, as well as cardiorespiratory disease morbidity and mortality (Brook et al. 2010; Hoek et al. 2013). Recently, association between long-term exposure to ambient air pollution, benzene and nitrogen dioxide, and lung cancer has been reported in North America and Europe (Puett et al. 2014; Raaschou-Nielsen et al. 2013; Villeneuve et al. 2014). Especially fine particulate matter [PM < 2.5 μm (PM_2.5_)] is believed to be responsible for the associations. The World Health Organization (WHO) estimates 3.7 million premature deaths worldwide in 2012 due to ambient air pollution (WHO 2014).

Findings based on animal models suggest that oxidative stress and inflammatory responses initiated upon deposition of fine PM in the alveoli may be key pathophysiologic mechanisms linking exposure to ambient fine particles to both respiratory and cardiovascular diseases in humans (Cassee et al. 2013). Oxidative stress and inflammation have also been proposed as underlying mechanisms linking PM and cancer, including lung cancer (Soberanes et al. 2012; Zhao et al. 2013). Despite these findings, the extent to which systematic effects are elicited by ambient particles, and the detailed pathways activated are still under debate (Peters 2012). Novel molecular approaches such as genome-wide methylation assays allow a hypothesis-free assessment of changes in the regulation of blood leukocytes, involved in CVD development (Baccarelli and Bollati 2009).

Changes in global methylation as well as in candidate genes (Bind et al. 2014) were observed in individuals with high-occupational exposure such as foundry workers in a small study (Tarantini et al. 2009) or in response to ambient PM concentrations a few hours before the study visit (Baccarelli et al. 2009). However it is difficult to determine the exact time window associated with methylation.

Genome-wide methylation assays allow taking advantage of advances in biological technologies in epidemiological studies (Christensen and Marsit 2011) and studying in particular the role of ambient fine particle concentrations in the days and weeks before biosample collection.

The objective of the analyses presented here is to identify and investigate DNA methylation at CpG (cytosine-guanine dinucleotide) sites in association with short- and mid-term PM_2.5_ ambient exposure. In addition, we consider biological pathways that might mediate associations between PM_2.5_ and health outcomes, based on the specific CpG sites identified.

## Methods

Three independent cohort studies formed the basis for the analyses presented here. Uniform methods were applied for fine particle measurements and methylation methods.

### Study Populations

KORA F3 and F4 cohorts are follow-up studies from the previous KORA S3 and S4, which enrolled all inhabitants of German nationality between the ages of 25 and 74 years old from the region of Augsburg, South Germany, in accordance with principles of the Declaration of Helsinki (World Medical Association Declaration of Helsinki 2008). They included 3,988 participants from F3 and 4,227 participants from F4; data were collected between 2004 and 2005 (F3) and between 2006 and 2008 (F4). Exhaustive information about these two studies has been described previously (Holle et al. 2005; Wichmann et al. 2005). Methylation profiles were evaluated for a total of 500 KORA F3 participants and 1,799 F4 participants. No sample overlap appears between F3 and F4. All participants supplied written informed consent that were approved by the Ethics Committee of the Bavarian Medical Association.

The Veteran Affairs (VA) Normative Aging Study (NAS) is an ongoing longitudinal study of aging, which began in 1963; details of this study have been published previously (Bell et al. 1972). Briefly, the NAS is a closed cohort of 2,280 male volunteers from the Greater Boston area who were 21–80 years old at entry. They were enrolled after an initial health screening determined that they were free of known chronic medical conditions. The present study was approved by the Department of Veterans Affairs Boston Healthcare System, and written informed consent was obtained from subjects prior to participation. The NAS participants have been reevaluated every 3–5 years using detailed on-site physical examinations and questionnaires. Blood samples were provided from 657 participants and for most of them a second sample was drawn (1,119 samples in total) between 1999 and 2007.

We restricted the current analysis to white participants (*n* = 657) in order to increase comparability across the three cohorts.

### Profiling of DNA Methylation

We used the Illumina 450k Beadchip (following the Illumina Infinium HD Methylation Protocol) to assess DNA methylation in more than 480,000 CG CpG methylation sites throughout the entire genome (Zeilinger et al. 2013). Detailed validation and evaluation of this technology are provided by Sandoval et al. (2011) and Dedeurwaerder et al. (2011). Outputs of the chip are beta values that represent the percentage of methylation for every CpG target. Since the microarray measures each CpG site with either of two technically distinct types of probes, the distribution of resulting methylation values differs. We used the following approach to preprocess the data: *a*) data quality: removal of records according to functional beads, detection *p*-value and SNP frequency; *b*) data correction: background subtraction and dye bias adjustment; *c*) probe type adjustment: beta-mixture quantile normalization (BMIQ; Teschendorff et al. 2013). Normalization process was chosen based on review papers (Marabita et al. 2013; Wu et al. 2014).

### Environmental Measurement

Specifically, in KORA, PM_2.5_ mass concentration in ambient air and temperature were measured hourly at one monitoring station approximately 1 km southeast of the city center of Augsburg for the entire study period from 2004 to 2008 (Birmili et al. 2010; Pitz et al. 2008) with the tapered element oscillating microbalance device (TEOM® series 1400A, Thermo Electron Corporation, East Greenbush, NY USA) as described by Patashnick and Rupprecht (1990). Forty-four days were missing in KORA in 2004–2008 and eventually excluded from calculation of trailing averages.

In NAS, ambient PM_2.5_ concentration was monitored in downtown Boston 1 km from the VA medical center. We measured hourly PM_2.5_ concentrations with the same device as in Augsburg. Hourly temperature data were obtained from the Boston Logan Airport (Boston, MA) weather station (12 km from the medical center). Sampling, processing of samples, analysis and reporting were conducted according to standard operating procedures (Dockery et al. 2005). Missing hourly concentration data for PM_2.5_ were imputed using regression modeling, including a long-term time trend, day of week, hour of day, temperature, relative humidity, barometric pressure, and nitrogen dioxide concentrations (NO_2_) as predictors.

### Statistical Analysis

An Epigenome-Wide Analysis Study (EWAS) was conducted in each of the three studies. Based on previous knowledge (Baccarelli et al. 2009; Brüske et al. 2010; Steenhof et al. 2014; Zeilinger et al. 2013) we defined *a priori* model with the following covariates: age, personal income (education years for NAS, in which information on income was not available), alcohol intake, BMI, temperature (trailing average always matching with the PM exposure window), and the proportion of five white blood cell types: monocytes, B Cells, CD8 T cells, CD4 T cells, NK [estimated with a method developed by Houseman et al. (2012)] as continuous variables; and sex, smoking status (never, former, current and passive-only for KORA-smokers), day of the week, and season (according to the astronomical definition) as categorical variables (Table 1). In order to investigate the association between short- and mid-term PM_2.5_ and DNA methylation, we considered three different averaging periods (2-, 7- and 28-day) backwards starting from the day of the visit, decided *a priori* based on Bind et al. (2014), Schwartz (2000) and Rückerl et al. (2007). For KORA, multivariable linear regression models (Equation 1) were used to investigate the association between PM_2.5_ exposure and methylation values:

**Table 1:** Descriptive statistics of the study participants in the KORA F3, KORA F4, and the U.S. Veteran Affairs Normative Aging Study (NAS).

Variables	Mean ± SD/N (%)		
KORA F3 (*n* = 500, 2004–2005)	KORA F4 (*n* = 1,799, 2006–2008)	NAS baseline^*a*^ (*n* = 657)
Participants characteristics
Males	260 (52.0)	887 (49.3)	657 (100)
Age (years)	53.12 ± 9.6	60.92 ± 8.9	72.44 ± 6.9
BMI (kg/m^2^)^*b*^	27.15 ± 4.5	28.15 ± 4.8	28.07 ± 4.1
Monthly Income (euros)	1104.8 ± 583.9	1159.84 ± 556.6	—
Education (years)	11.7 ± 2.8	11.5 ± 2.5	15.07 ± 2.9
Drinkers^*c*^	296 (59.2)	1,038 (57.7)	130 (19.7)
Alcohol consumption (g/day)	16.11 ± 19.6	15.49 ± 20.4	—
Smoking
Never smokers	226 (45.2)	226 (12.6)	188 (28.6)
Former smokers	11 (2.2)	782 (43.5)	446 (67.9)
Current smokers	232 (46.0)	753 (41.9)	23 (3.5)
Passive smokers (either former or never)	11 (2.2)	36 (2.0)	—
Missing	20 (4.4)	2 (0.0)	0 (0.0)
Environmental exposure (mean of the daily average of the day before the visit)
PM_2.5_ (μg/m^3^)^*d*^	20.0 ± 11.6	14.2 ± 10.2	10.6 ± 7.1
Percentiles (25th, 50th, 75th)	14.0, 17.7, 25.9	6.7, 12.2, 18.8	6.3, 9.0, 13.2
Temperature (°C)	7.1 ± 7.5	8.7 ± 6.6	12.5 ± 8.5
Percentiles (25th, 50th, 75th)	0.9, 7.9, 13.2	3.9, 7.5, 13.1	6.4, 12.7, 19.8
—, data not available. ^***a***^First time blood sample was collected (time window: 1999–2007). ^***b***^Body mass index. ^***c***^Participants with at least 2 drinks per week. ^***d***^PM < 2.5 μm.			

*Y_i_* = β*_0_* + β*_1_* PM*_2.5i_* + β*_2_* Temperature*_i_* + β*_3_ X_3i_* + … + β*_p_X_pi_* + ε*_i_*, [1]

where *Y_i_* is the methylation measurement for subject *i*, β*_0_* is the intercept, β*_1_* and β*_2_* are the coefficients of the trailing average values for exposure and temperature during the specific time window, *X_3i_* to *X_pi_* are the *p–2* covariates and ε*_i_* is the error. Effect estimates represent the difference in methylation associated with a 10 μg/m^3^ increase in PM_2.5_. For NAS data, we fitted generalized mixed-effect models (Equation 2) in order to account for the repeated measurements; time-variant covariates were assessed at both first and second visit and a random participant effect (*u_i_*) was applied in order to take the data collection at two different time points into account:

*Y_it_* = β*_0t_* + β*_1_*PM*_2.5it_* + β*_2_*Temperature*_it_* + β*_3_X_3it_* + … + β*_p_X_pit_* + *u_i_* + ε*_it_* [2]

Finally, we pooled cohort-specific estimates, when available for all three studies, for each exposure window by random-effect meta-analysis (428,415 CG targets). Bonferroni threshold (fixed at 7.5E-08) and false discovery rate [FDR, (Benjamini and Hochberg 1995)] with Benjamini-Hochberg criterion was used to adjust fixed-effect *p*-values for multiple comparisons. I-squared test on fixed-effect estimates have been used to assess heterogeneity and CpGs with *p*-values > 0.05 and *I* ^2^ < 0.5 were labeled as homogenous. Finally, a number of sensitivity analyses were performed. We repeated the *a priori* models with additional adjustment for average annual exposure during the year before the visit to assess potential confounding by long-term exposure. In addition, we ran models adjusted only for age and sex, and models adjusted only for age, sex, and white blood cell proportions. All analyses were performed using statistical software R (version 2.14; R Core Team 2014). Residual plots of significant CpGs were used to check whether the identified CpGs where driven by outliers. To discard these values, we used a rule of thumb based on biological knowledge. DNA methylation in the 0–1 range can be divided in hypo-, hemi- and hyper-methylation with ranges [0–0.35], [0.35–0.65] and [0.65–1] respectively. Once we selected the CpGs with very high residuals (absolute value above 0.25), we identified the methylation segment where the mean was located and discarded all the values out of it; we repeated the analysis.

Functional analysis of the identified genes has been performed via a web interface (Warde-Farley et al. 2010).

## Results

Data from three independent cohort studies were available (Table 1). Specifically, cross-sectional data from two independent subsamples of the KORA study (KORA F3, *n* = 500 participants; F4, *n* = 1,799) and cohort data collected as part of the NAS (*n* = 657) formed the basis of the analyses presented here. The NAS included only men with an average age of 72 years, while KORA F3 and F4 participants (52% and 49% of males) were on average 53 and 61 years old. While body mass index was rather similar, substantial differences were observed for years of education (mean of 15.1 in NAS versus 11.7 and 11.5 in KORA F3 and F4, respectively) and alcohol consumption (19.7% of drinkers for NAS versus 59.2% and 57.7% for KORA F3 and F4, respectively). Regarding smoking, KORA F3 consisted mostly of never and current smokers, KORA F4 of former and current, whereas approximately two-thirds of NAS participants were former smokers. The NAS, on average, had lower particle concentration the day before the visit but had higher average temperatures than did the KORA studies. During the study period, PM_2.5_ exceeded the daily U.S. Environmental Protection Agency (EPA) standard of 35 μg/m^3^ 7.5% of the days in F3 (2004–2005), 5.9% in F4 (2006–2008) and 2.9% in NAS (1999–2007) (U.S. EPA 2004). Consistent methylation averages were observed between the three studies with relatively small standard deviations (Tables 2–3).

**Table 2:** Characteristics of the CpG sites from meta-analyses of 2- and 7-day trailing averages, significant with Bonferroni or FDR methods.

Name	CHR	Reference gene name	Methylation level Illumina Beta, mean ± SD				Fixed-effect regression coefficient^*a*^	Sig.^*b*^	FDR^*c*^	*I*^2^ (%)	Sig. *I*^2^
F3	F4	NAS	Mean
Trailing 2-day average PM_2.5_^*d*^
cg25575464	17	*NEURL4*	0.03 ± 0.01	0.02 ± 0.01	0.01 ± 0.01	0.02 ± 0.01	0.00082	4.69E-08	0.005	91.0	< 0.001
Trailing 7-day average PM_2.5_^*e*^
cg04078416	3	*CCDC12*	0.05 ± 0.01	0.05 ± 0.01	0.02 ± 0.01	0.04 ± 0.01	0.0001	4.19E-07	0.027	0.0	0.52
cg15996282	5	*LMBRD2*; *SKP2*	0.04 ± 0.01	0.04 ± 0.03	0.02 ± 0.01	0.04 ± 0.02	0.0020	7.25E-07	0.010	0.0	0.55
cg00402617	8	*YWHAZ*	0.07 ± 0.01	0.06 ± 0.02	0.03 ± 0.01	0.06 ± 0.02	0.0002	1.29E-07	0.018	62.3	0.07
cg19963313^*f*^	8	*NSMAF*	0.04 ± 0.01	0.03 ± 0.01	0.02 ± 0.01	0.03 ± 0.01	0.0018	2.49E-08	0.016	0.0	0.59
cg15883382	10	NA^*g*^	0.04 ± 0.01	0.05 ± 0.01	0.02 ± 0.01	0.04 ± 0.01	0.0001	8.43E-07	0.040	62.2	0.07
cg09225537	15	*MAG*	0.03 ± 0.01	0.02 ± 0.01	0.01 ± 0.01	0.02 ± 0.01	0.0001	4.44E-07	0.027	0.0	0.75
cg08757611	17	NA^*g*^	0.03 ± 0.01	0.03 ± 0.01	0.02 ± 0.01	0.03 ± 0.01	9.70E-05	2.15E-07	0.018	0.0	0.68
cg25575464	17	*NEURL4*	0.03 ± 0.01	0.02 ± 0.01	0.01 ± 0.01	0.02 ± 0.01	0.0001	1.76E-07	0.018	87.6	< 0.001
cg02608596^*f*^	19	*MPND*	0.04 ± 0.01	0.03 ± 0.02	0.02 ± 0.01	0.03 ± 0.02	0.0017	7.69E-08	0.010	4.8	0.35
CHR, chromosome; FDR, False discovery rate. ^***a***^Estimated difference in methylation for a 10-μg/m^3^ increase in PM_2.5_ adjusted for sex, age, income (education years for NAS, in which information on income was not available), smoking status, alcohol intake, body mass index, temperature (moving average always matching with the PM exposure window), day of the week, season and the proportion of five estimated white blood cell types: monocytes, B cells, CD8 T cells, CD4 T cells, NK. ^***b***^*p*-Values, Bonferroni significance level at 7.5E-08. ^***c***^FDR using Benjamini-Hochberg (1995) method, significance level at 0.05. ^***d***^2-day trailing average starting from the day of the visit. ^***e***^7-day trailing average starting from the day of the visit. ^***f***^Shown in Figure 2. ^***g***^NA: no annotated gene.											

**Table 3:** Characteristics of the CpG sites from meta-analysis of 28-day trailing average, significant with Bonferroni method, or FDR significant and located in a gene with another CpG that meets genome-wide significance, or FDR significant and Bonferroni significant at shorter time-window.

Name	CHR	Reference gene name	Methylation level Illumina Beta, mean ± SD				Fixed-effect regression coefficient^*a*^	Sig.^*b*^	FDR^*c*^	*I*^2^ (%)	Sig. *I*^2^
F3	F4	NAS	Mean
cg16308101	1	*SERBP1*	0.45 ± 0.03	0.46 ± 0.03	0.44 ± 0.03	0.45 ± 0.03	–0.0076	2.86E-08	0.002	91.3	< 0.001
cg16856342^*d*^	1	*SERBP1*	0.46 ± 0.02	0.46 ± 0.02	0.38 ± 0.02	0.44 ± 0.02	–0.0061	1.74E-07	0.003	1.4	0.36
cg23276912^*e*^	1	*C1orf212*	0.87 ± 0.03	0.89 ± 0.03	0.86 ± 0.04	0.90 ± 0.03	0.0073	4.56E-08	0.002	25.9	0.26
cg03455255	2	*TSPYL6*; *ACYP2*	0.90 ± 0.02	0.92 ± 0.01	0.93 ± 0.02	0.92 ± 0.02	0.0047	1.86E-08	0.001	61.8	0.073
cg11046593^*e*^	2	*MSGN1*	0.80 ± 0.05	0.83 ± 0.09	0.86 ± 0.07	0.83 ± 0.08	0.016	1.12E-08	0.001	46.1	0.16
cg04423572	3	*ACVR2B-AS1*	0.70 ± 0.04	0.74 ± 0.04	0.74 ± 0.03	0.73 ± 0.04	0.013	7.26E-09	0.001	97.3	< 0.001
cg19963313^*f*^	8	*NSMAF*	0.04 ± 0.01	0.03 ± 0.01	0.02 ± 0.01	0.03 ± 0.01	0.0024	4.12E-07	0.005	0.0	0.90
cg13169286	10	NA^*g*^	0.55 ± 0.03	0.59 ± 0.07	0.51 ± 0.06	0.57 ± 0.06	–0.013	6.21E-08	0.003	85.4	< 0.001
cg02795981^*d*^	10	*ZMIZ1*	0.78 ± 0.05	0.78 ± 0.06	0.79 ± 0.08	0.78 ± 0.06	0.0093	3.94E-05	0.029	49.5	0.14
cg19215199	10	*ZMIZ1*	0.82 ± 0.04	0.83 ± 0.04	0.82 ± 0.06	0.83 ± 0.04	0.0093	3.66E-08	0.002	94.3	< 0.001
cg13527922	11	*F2*	0.86 ± 0.02	0.87 ± 0.02	0.87 ± 0.02	0.87 ± 0.02	0.0051	1.54E-08	0.001	81.9	0.004
cg24101979^*d*^	17	*NXN*	0.81 ± 0.03	0.77 ± 0.04	0.80 ± 0.05	0.78 ± 0.04	0.0072	8.95E-05	0.001	92.4	< 0.001
cg26003785^*e*^	17	*NXN*	0.94 ± 0.01	0.96 ± 0.01	0.97 ± 0.02	0.96 ± 0.01	0.0038	9.53E-09	0.001	27.3	0.25
cg26283240^*d*^	17	*NXN*	0.87 ± 0.03	0.86 ± 0.03	0.88 ± 0.04	0.87 ± 0.03	0.0065	2.03E-05	0.024	85.6	< 0.001
cg06004017^*d*^	22	*MN1*	0.86 ± 0.02	0.90 ± 0.02	0.87 ± 0.03	0.89 ± 0.02	0.0046	0.00019	0.048	74.6	0.02
cg20680669	22	*MN1*	0.96 ± 0.02	0.96 ± 0.02	0.99 ± 0.01	0.97 ± 0.02	–0.0049	2.09E-08	0.001	67.4	0.046
CHR, chromosome; FDR, false discovery rate. ^***a***^Estimated difference in methylation for a 10-μg/m^3^ increase in PM_2.5_ adjusted for sex, age, income (education years for NAS, in which information on income was not available), smoking status, alcohol intake, body mass index, temperature (moving average always matching with the PM exposure window), day of the week, season, and the proportion of five estimated white blood cell types: monocytes, B cells, CD8 T cells, CD4 T cells, NK. ^***b***^*p*-Values, Bonferroni significance level at 7.5E-08. ^***c***^FDR with Benjamini-Hochberg (1995) method, significance level at 0.05. ^***d***^Non-Bonferroni significant but FDR significant CpGs located in the a gene with a Bonferroni significant CpG. ^***e***^Shown in Figure 3. ^***f***^FDR significant and Bonferroni significant at 7-day PM_2.5_. ^***g***^NA: no annotated gene. Note: 28-day Trailing average starts from the day of the visit. A complete list of all CpGs that meet genome-wide significance or FDR significance for 28-day PM_2.5_ is provided in Excel File S1.											

**Figure 1:**
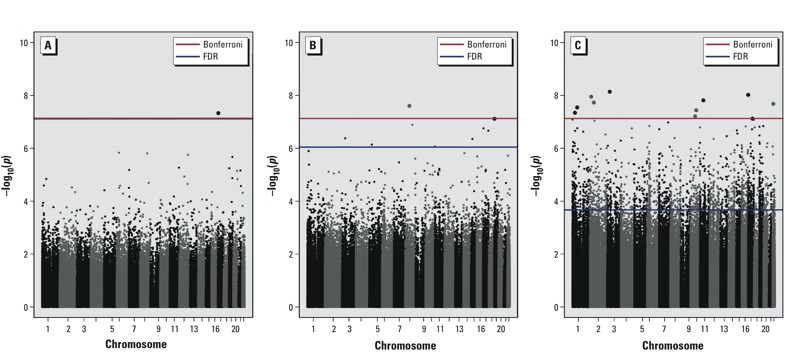
Manhattan plots showing fixed-effect *p*-values from the meta-analysis of KORA F3, KORA F4, and NAS longitudinal cohort studies across the human genome after fully adjusted model. Each dot corresponds to a CpG methylation site. Panel A: 2-day PM_2.5_ exposure; Panel B: 7-day PM_2.5_ exposure; Panel C: 28-day PM_2.5_ exposure (μg/m^3^).

**Figure 2:**
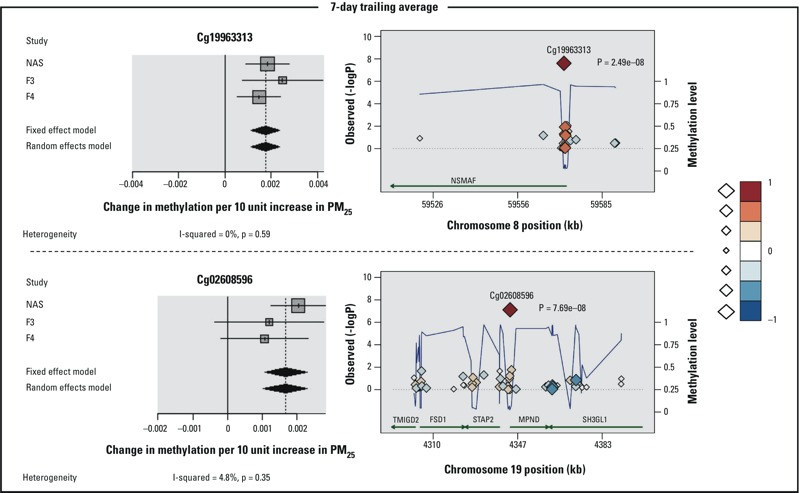
Forest plots (left side) and regional plots regarding cg19963313 that achieved genome-wide significance level and cg02608596 that was close to genome-wide significance at 7-day average and showed homogeneity. Forest plots show KORA F3, KORA F4, and NAS longitudinal cohort estimates and pooled meta-analysis results. Regional plots show the *p*-values from Figure 1, Panel B of each annotated CpG sites (diamonds) in a 200k bp length genome segment around the top CpG. The color and the size of the diamonds represent the intensity of the correlation with the top CpG target (in the center). The blue broken line connects the average methylation value of adjacent CpG sites; the right axis displays the 0–1 methylation scale. Correlations and averages values are calculated as mean of the three studies. Green arrows represent gene extension.

**Figure 3:**
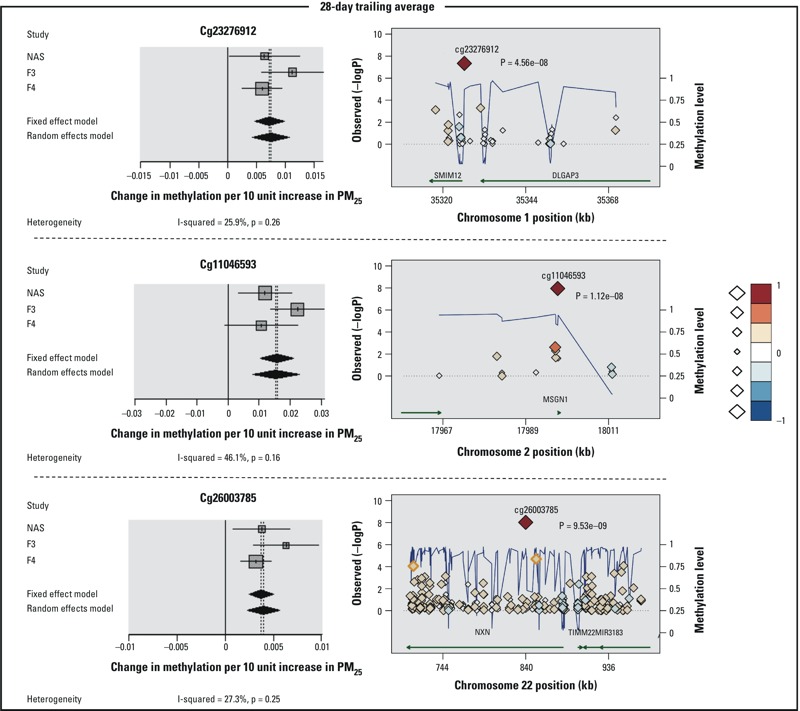
Forest plots (left side) and regional plots regarding the CpGs that achieved Bonferroni genome-wide significance level and homogeneity at 28-day exposure. Forest plots show KORA F3, KORA F4 and NAS longitudinal cohort estimates and pooled meta-analysis results. Regional plots show the *p*-values from Figure 1, Panel C of each annotated CpG sites (diamonds) in a 200k bp length genome segment around the top CpG. The color and the size of the diamonds represent the intensity of the correlation with the top CpG target (in the center). The blue broken line connects the average methylation value of adjacent CpG sites; the right axis displays the 0–1 methylation scale. Correlations and averages values are calculated as mean of the three studies. Green arrows represent gene extension. Orange outlined diamonds highlight FDR significant CpG sites.

The meta-analyses identified genome-wide significant (*p* < 7.5E-08) associations between PM_2.5_ exposure averaged over 2 days up to 4 weeks and single CpG-sites (Figure 1). DNA methylation at one CpG site (cg25575464 within *NEURL4*, chromosome 17) reached genome-wide significance (*p* < 7.5E-08) in association with 2-day trailing average PM_2.5_, with a positive association indicating higher methylation at 10-μg/m^3^ increase in exposure (Table 2; see also Figure S1). Although study-specific associations were all positive, there was significant heterogeneity among the studies. For 7-day average PM_2.5_ concentration, the association with one CpG site, cg19963313 (*NSMAF*, chr 8) reached genome-wide significance (Table 2), and study-specific estimates were positive and homogenous (*I* ^2^ = 0.0, *p*-value 0.59) (Figure 2). Associations between 7-day PM_2.5_ and cg02608596 (*MPND*, chr 9) also were positive and homogeneous among the three studies, but the *p*-value was slightly above the alpha level for genome-wide significance (*p* = 7.69E–08). Cg02608596 and 7 additional CpGs had FDR *p*-values < 0.05 for 7-day PM_2.5_, including cg25575464, which also was associated with 2-day PM_2.5_ (Table 2). Associations between 7-day PM_2.5_ and the additional CpGs were heterogeneous among the study sites in three cases and homogeneous in four cases. No additional CpG sites were identified as associated with 2-day PM_2.5_ based on FDR < 0.05. Associations between 10 CpGs and 28-day average exposure to PM_2.5_ reached genome-wide significance, including 3 with lower methylation [cg16308101 (*SERBP1*, chr 1), cg13169286 (no annotated gene, chr 10), and cg20680669 (*MN1*, chr 22)] and 7 with higher methylation [cg23276912 (*C1orf212*, chr 1), cg03455255 (*TSPYL6*, *ACYP2*, chr 2), cg11046593 (*MSGN1*, chr 2), cg04423572 (*ACVR2B-AS1*, chr 3), cg19215199 (*ZMIZ1*, chr 10), cg13527922 (*F2*, chr 11), cg26003785 (*NXN*, chr 17)] (Table 3). Study-specific associations were homogenous for cg23276912, cg11046593, and cg26003785 (Figure 3), but heterogeneous for the other CpGs (see Figure S1). When we considered all associations with FDR *p* < 0.05, a total of 1,829 CpG sites were associated with 28-day average PM_2.5_ (see Excel File S1), including 5 in genes with at least one Bonferroni significant CpG (also shown in Table 3): cg16856342 (*SERBP1*, chr 1), cg02795981 (*ZMIZ1*, chr 10), cg24101979 and cg26283240 (*NXN*, chr 17) and cg06004017 (*MN1*, chr 22). One CpG with a significant FDR for 28-day PM_2.5_ reached genome-wide significance for 7-day PM_2.5_ (cg19963313, *NSMAF*, chr 8).

### Sensitivity Analysis

Genome-wide significant CpGs at 28-day were also adjusted for long-term exposure and resulted in consistent estimates and *p*-values, except for cg20680669 which estimate moved from a ß = –0.0049 with *p* = 2.09E-08 (without long-term) to β = –0.0020 with *p* = 2.36E-03 and cg26003785 which moved from β = 0.0038 with *p* = 9.53E-09 to β = 0.0033 with *p* = 1.10E-06 (see Table S2). Furthermore, we checked for potential influences of outliers (see Figures S2–S4). Cg11046593 was of concern in these plots and 22 values were excluded for F4, 1 for F3 and 12 for NAS. However, the association remained significant: the estimate moved from 0.016 to 0.012 and the *p*-value from 1.12E-08 to 5.48E-08.

## Discussion

This meta-analysis of three cohort studies identified 12 CpGs genome-wide significantly associated with ambient fine particulate matter concentrations at different exposure times based on Bonferroni corrections. Based on previous knowledge (Bind et al. 2014; Rückerl et al. 2007; Schwartz 2000), we considered three different cumulative exposure windows: 2, 7 and 28 days, and we observed that the number of associations was larger for the longest exposure window. Nine CpG sites displayed increased methylation and three decreased methylation after exposure to fine ambient particle concentrations. All identified methylation sites displayed little overall variation (average co-efficient of variation was 15%) within the study populations. Four of them manifested homogeneous changes across the three different studies. Applying FDR, 7 and 1,819 additional CpGs were found significant at 7- and 28-day average PM_2.5_ exposure, respectively.

The CpG site (cg19963313) identified with the 7-day trailing average showed homogeneity among the studies. Cg19963313 is positioned in the gene *NSMAF* that is linked with the 55kD tumor necrosis factor receptor since it encodes a WD-repeat protein that binds its cytoplasmic sphingomyelinase activation domain (Montfort et al. 2010). Moreover, it participates in the same reaction within a pathway as *SMPD2* (Wu et al. 2010), which has been demonstrated in primary cells to be linked to oxidative stress (Byon et al. 2008; Jana and Pahan 2007). Furthermore, it has been identified in cellular response to hyperosmolar stress (Robciuc et al. 2012). Hyperosmolarity is well known to impose remarkable stress on membranes, especially the ones that are in direct contact with the environment (Hallows et al. 1996), but it has never been associated with air pollution.

Furthermore we identified three CpG sites significantly and homogeneously associated with the 28-day average exposure to fine particle: cg26003785, cg11046593 and cg23276912 annotated to *NXN*, *MSGN1* and *C1orf212,* respectively, which are protein-coding genes.

Specifically, *NXN* has been observed as partner of phosphofructokinase (PFK) 1, a glycolytic enzyme, reported as contributor for systemic metabolic conditions and also cancerous processes (Mor et al. 2011; Yi et al. 2012).

Increased methylation was detected at cg11046593, located in the promoter of *MSGN1*, that—when methylated—has been shown to lead to transcriptional repression (Jones and Takai 2001). Domain databases also determined shared protein domain with *AHR* (aryl hydrocarbon receptor) and *ARNT* (aryl hydrocarbon receptor nuclear translocator), involved in regulation of inflammatory processes implicated in multi-factorial diseases like pulmonary disorders (Scrivo et al. 2011; Ukena et al. 2010). It was found that these two genes regulate chemokine-responses mostly relating *AHR* and *ARNT* to the nuclear factor-κB family (NF-κB) where the p65/p50 dimer is pivotal in the regulation of the inflammatory responses (Øvrevik et al. 2014; Vogel and Matsumura 2009). *AHR* and particulate matter exposure have already been associated through nongenotoxic events and Th17 polarization (Andrysik et al. 2011; van Voorhis et al. 2013), but here we observed an epigenetic factor as possible mediator. Even without a direct association, the discovery of *MSGN1* provides a novel hypothesis in the path between exposure to endogenous factors and immunological system responses and future studies are needed to verify and eventually clarify the possible role of *ARNT*.

### Temporal Variation within Short- and Mid-Term Range

For cumulative exposures over 28 days, 10 CpG sites were genome-wide significant. Larger datasets are needed to better understand the optimal exposure time window and to confirm a hypothesis, that it may be CpG site-specific. The cases of cg25575464 (Bonferroni significant at 2-day, FDR significant at 7-day and non-significant at 28-day average) and cg19963313 (non-significant at 2-day, Bonferroni significant at 7-day and FDR significant at 28-day average), might be consistent with the hypothesis regarding CpGs associated at shorter time periods but not over longer time.

One of the genes we highlighted, *ZIMZ1*, has already been connected to skin tumors in mouse models (Rogers et al. 2013) and our results, independently, link it to PM exposure via DNA methylation, reinforcing the hypothesized role of epigenetics in the pathways to tumor development (Laird 2005).

We observed mostly positive effect estimates, in this genome-wide methylation study, in contrast with previous results (Guo et al. 2014) that observed a negative association between short-term PM exposure and DNA methylation in tandem repeats. Zeilinger et al. (Zeilinger et al. 2013) observed decreased methylation as consequence of active smoking in a cross-sectional study. Their most striking and significant CpG belongs to *AHRR* (aryl hydrocarbon receptor repressor) that repress *AHR*, and we observed increased methylation in a gene that shares protein domain with *AHR*. Possible relations and implications need to be verified in the future.

### Strengths and Limitations

The data presented here combines evidence from three independent studies, each considering data of at least 500 participants, a paramount element to identify differentially methylated CpG sites that have very little variability. We also adjusted our models for important variables that may otherwise confound the effect of associations with ubiquitous exposures such as ambient air pollution. Finally, we used daily averages of temperature to calculate the same trailing averages and apply appropriate adjustment for weather conditions. We performed a number of sensitivity analyses. Overall, the results of *a priori* chosen model were considered a conservative estimate. The observed hits between PM_2.5_ and CpG sites were independent of long-term exposure at the residence and were not influenced by potential outliers. This study has also limitations. There is a consensus in the scientific community that a background station measuring particulate matter with aerodynamic diameter ≤ 2.5 μm (PM_2.5_) mass concentrations could be regarded as representative for larger urban areas (Monn 2001). Considering that no coal power plant is in operation in proximity of the participants and only a small percentage of them live close to a major road we had to rely on ambient air pollution measurements since personal exposures were not available. Measurement error from using a single site in this study is expected to result in primarily Berkson-type measurement error (Zeger et al. 2000), which would bias the standard errors, but not the estimated associations. We also acknowledge that the study included only whites, and generalizability to other populations is uncertain. While KORA was cross-sectional, the NAS study assessed the role of ambient particles longitudinally on time. Nevertheless, we had no comparable exposure estimates available to assess the long-term effect of ambient particles. Finally, the Illumina 450k does not completely cover the entire epigenome.

## Conclusions

In conclusion, in this epigenome-wide investigation of CpG dinucleotide methylation, we highlighted several CpG sites associated with cumulative exposure to ambient particles up to a month. The trend of significance level of our results tends to increase with the length of the averaging period and the majority shows an increase in methylation. The identified genetic loci suggest novel biological pathways that may link ambient particulate matter to health outcomes such as tumor development and also gene regulation, inflammatory stimuli, pulmonary disorders and glucose metabolism. Future mechanistic studies are needed to establish whether these epigenetic changes could potentially explain the evidence found for ambient fine particles and lung cancer incidence.

## Supplemental Material

(1.3 MB) PDF

(116 KB) ZIP
