# Supplementary material for: Genome-Wide Analysis of DNA Methylation and Fine Particulate Matter Air Pollution in Three Study Populations: KORA F3, KORA F4, and the Normative Aging Study
Source: Environ Health Perspect. 2016 Jan 5;124(7):983–90. doi: 10.1289/ehp.1509966 (PMC4937859; doi:10.1289/ehp.1509966)
Supplement: (1.3 MB) PDF [file ehp.1509966.s001.acco.pdf]

Environ Health Perspect
DOI: 10.1289/ehp.1509966

Note to readers with disabilities: *EHP* strives to ensure that all journal content is accessible to all readers. However, some figures and Supplemental Material published in *EHP* articles may not conform to 508 standards due to the complexity of the information being presented. If you need assistance accessing journal content, please contact ehp508@niehs.nih.gov. Our staff will work with you to assess and meet your accessibility needs within 3 working days.

Supplemental Material

A Genome-Wide Analysis of DNA Methylation and Fine Particulate Matter Air Pollution in Three Study Populations: KORA F3, KORA F4, and the Normative Aging Study

Tommaso Panni, Amar J. Mehta, Joel D. Schwartz, Andrea A. Baccarelli, Allan C. Just, Kathrin Wolf, Simone Wahl, Josef Cyrys, Sonja Kunze, Konstantin Strauch, Melanie Waldenberger, and Annette Peters

Table of Contents

Figure S1. Forest plots for the eight CpG sites that showed Bonferroni genome-wide significance but results heterogeneous (I^2 p-value<0-05 or $I^2 > 0.5$).

Figure S2. Residual plot for FDR significant CpG at 2-day average (F3=blue, F4=red, NAS=green).

Figure S3. Residual plot for FDR significant CpG at 7-day average (F3=blue, F4=red, NAS=green).

Figure S4. Residual plot for Bonferroni significant CpG at 28-day average (F3=blue, F4=red, NAS=green). For cg11046593, 21 methylation values were excluded and re-run the models and the meta-analysis. New p-value resulted 5.48E-08, still Bonferroni significant.

Table S1. Study specific regression coefficients of FDR (2- and 7-day average) and Bonferroni (28-day average) significant CpGs.

Table S2. Sensitivity analysis, comparison of fixed-effect regression coefficients (ß) and p-values of fully-adjusted models in 28-day significant hits with and without yearly $PM_{2.5}$ exposure adjustment.

Supplemental Code and Data Zip File

Excel File S1. FDR significant CpG sites for 28-day $PM_{2.5}$

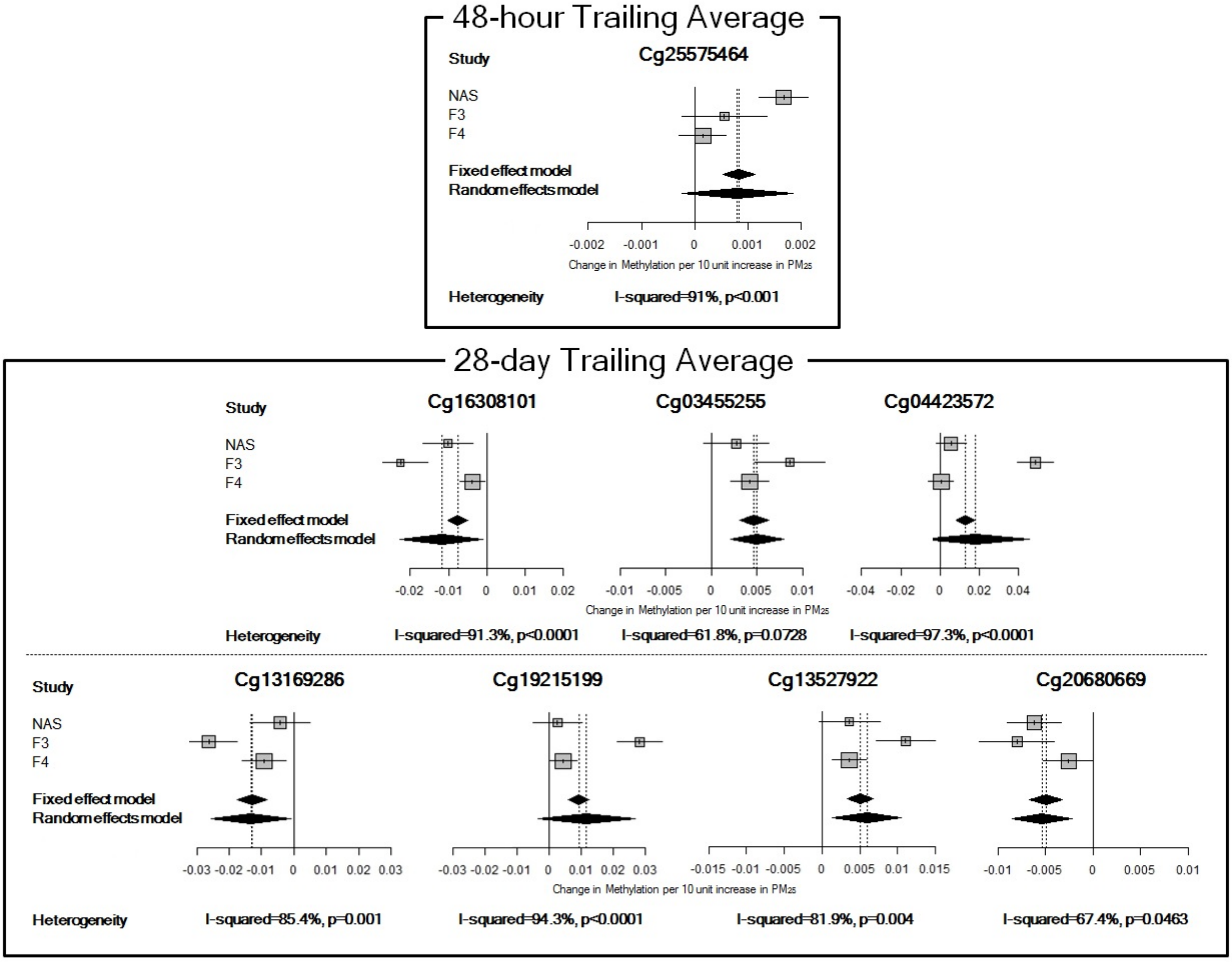

Figure S1. Forest plots for the eight CpG sites that showed Bonferroni genome-wide significance but results heterogeneous (I^2 p-value<0-05 or I^2 > 0.5).

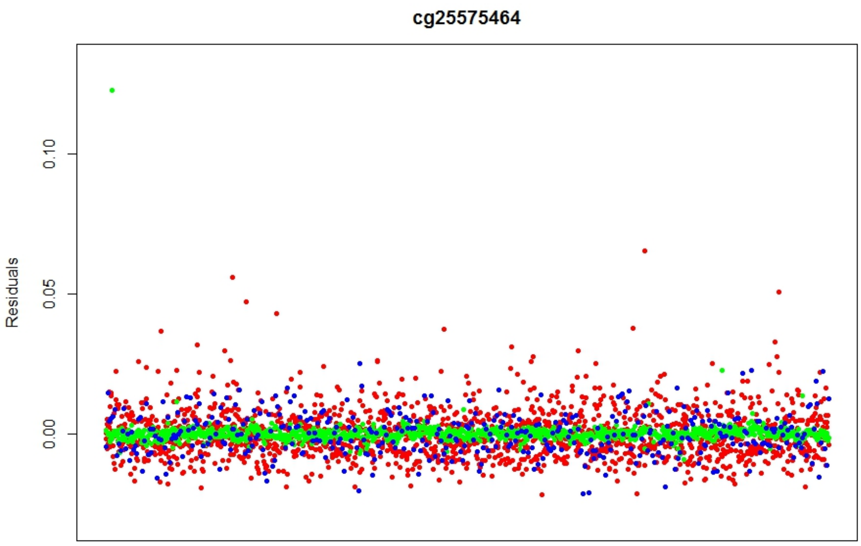

Figure S2. Residual plot for FDR significant CpG at 2-day average (F3=blue, F4=red, NAS=green).

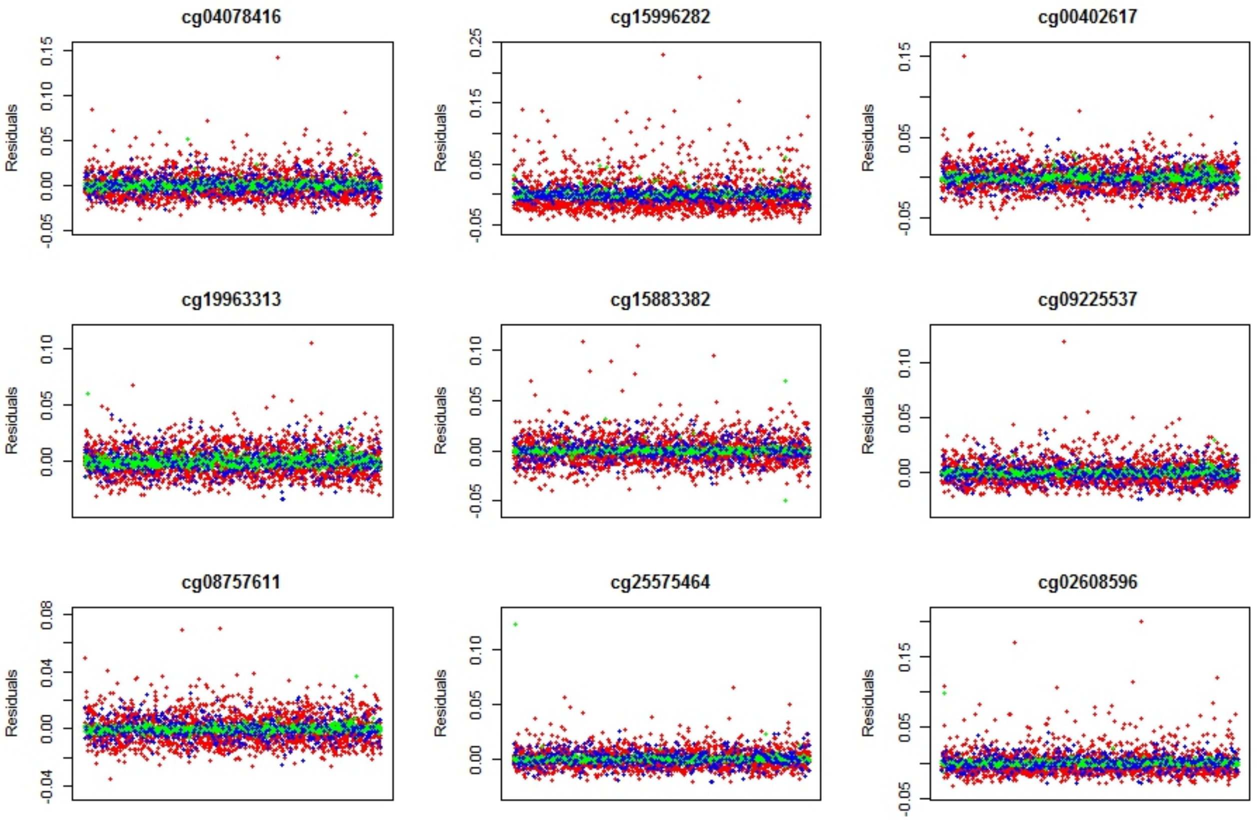

Figure S3. Residual plot for FDR significant CpG at 7-day average (F3=blue, F4=red, NAS=green).

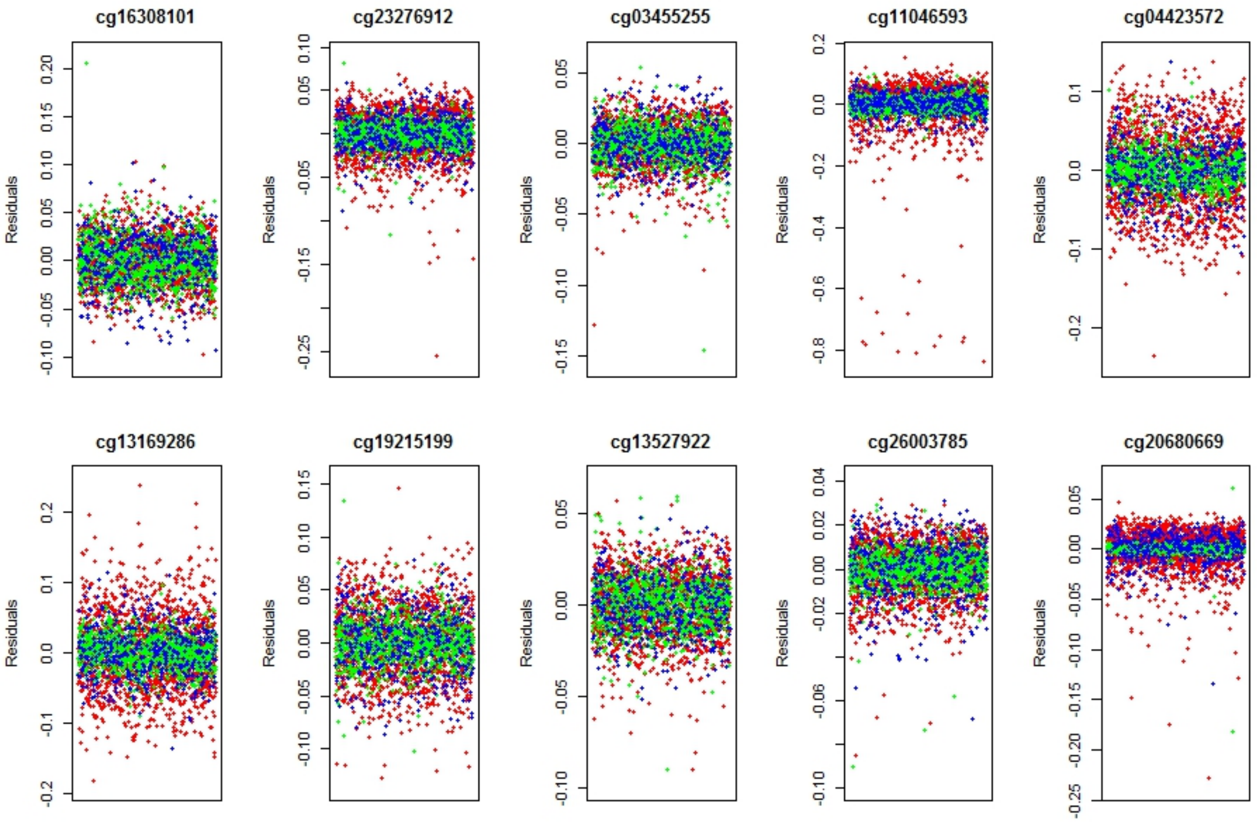

Figure S4. Residual plot for Bonferroni significant CpG at 28-day average (F3=blue, F4=red, NAS=green). For cg11046593, 21 methylation values were excluded and re-run the models and the meta-analysis. New p-value resulted 5.48E-08, still Bonferroni significant.

Table S1. Study specific regression coefficients of FDR (2- and 7-day average) and Bonferroni (28-day average) significant CpGs.

CpG	NAS		F3		F4	
	**ß***	**P-value**	**ß**	**P-value**	**ß**	**P-value**
**2-day Trailing Average**						
**cg25575464†**	0.00167	6.83E-12	5.58E-04	0.17597	1.51E-04	0.502384
**7-day Trailing Average**						
**cg00402617**	0.00169	0.01372	0.00422	2.65E-05	0.00174	0.002803
**cg02608596**	0.00204	5.02E-07	0.0012	0.138056	0.00106	0.096751
**cg04078416**	0.00129	0.000277	8.52E-04	0.266829	0.00184	0.000476
**cg08757611**	9.30E-04	5.95E-05	6.43E-04	0.279705	0.00122	0.001554
**cg09225537**	0.00116	9.71E-05	0.00148	0.02347	9.22E-04	0.020167
**cg15883382**	0.00295	4.15E-06	0.00105	0.181377	0.00125	0.013804
**cg15996282**	0.0023	3.34E-05	0.00183	0.008537	0.00108	0.279726
**cg19963313†**	0.00183	0.000141	0.00249	0.005548	0.00147	0.001984
**cg25575464**	0.00248	7.86E-10	4.82E-04	0.390485	5.65E-04	0.07276
**28-day Trailing Average**						
**cg03455255**	0.00271	0.140615	0.00857	1.81E-05	0.0042	7.24E-05
**cg04423572**	0.00564	0.15232	0.04859	4.58E-22	5.25E-04	0.872754
**cg11046593**	0.01191	0.007323	0.02234	5.85E-07	0.01072	0.072973
**cg13169286**	-0.0043	0.360508	-0.0263	6.31E-09	-0.0092	0.009082
**cg13527922**	0.0036	0.081134	0.01107	7.09E-08	0.00359	0.001815
**cg16308101**	-0.0101	0.002248	-0.0225	1.13E-09	-0.0038	0.020929
**cg19215199**	0.0027	0.49826	0.0283	5.52E-14	0.00441	0.04335
**cg20680669**	-0.0061	2.95E-05	-0.008	7.67E-05	-0.0026	0.053071
**cg23276912**	0.00639	0.038665	0.01122	5.64E-05	0.00596	0.000643
**cg26003785**	0.00378	0.012775	0.00635	0.000282	0.00319	6.62E-05

* ß express change in %5mC associated with a 10 µg/m^3 increase in $PM_{2.5}$

† Bonferroni Significant

Table S2. Sensitivity analysis, comparison of fixed-effect regression coefficients (ß) and p-values of fully-adjusted models in 28-day significant hits with and without yearly $PM_{2.5}$ exposure adjustment.

	**With long-term**		**Without long-term**	
	**ß***	**P-value**	**ß***	**P-value**
**cg16308101**	-0.0070	4.64E-07	-0.0076	2.86E-08
**cg23276912**	0.0079	8.85E-09	0.0073	4.56E-08
**cg03455255**	0.0050	5.37E-09	0.0047	1.86E-08
**cg11046593**	0.016	1.28E-07	0.016	1.12E-08
**cg04423572**	0.013	1.23E-08	0.013	7.26E-09
**cg13169286**	-0.014	5.97E-08	-0.013	6.21E-08
**cg19215199**	0.0095	4.32E-08	0.0093	3.66E-08
**cg13527922**	0.0056	2.09E-09	0.0051	1.54E-08
**cg26003785**	0.0033	1.10E-06	0.0038	9.53E-09
**cg20680669**	-0.0020	2.36E-03	-0.0049	2.09E-08

* ß express change in %5mC associated with a 10 $\mu g/m^3$ increase in $PM_{2.5}$

Covariates: age, personal income (education years for NAS, in which information on income was not available), alcohol intake, BMI, temperature (moving average always matching with the PM exposure window) and the proportion of five white blood cell types: monocytes, B Cells, CD8 T cells, CD4 T cells, NK as continuous and sex, smoking status (never, former, current and passive - only for KORA - smokers), day of the week and season as categorical
